# Derivation of Arbas Cashmere Goat Induced Pluripotent Stem Cells in LCDM with Trophectoderm Lineage Differentiation and Interspecies Chimeric Abilities

**DOI:** 10.3390/ijms241914728

**Published:** 2023-09-29

**Authors:** Fang Liu, Jing Wang, Yongli Yue, Chen Li, Xuemin Zhang, Jinzhu Xiang, Hanning Wang, Xueling Li

**Affiliations:** State Key Laboratory of Reproductive Regulation and Breeding of Grassland Livestock, School of Life Sciences, Inner Mongolia University, Hohhot 010070, China; liuf185@163.com (F.L.);

**Keywords:** iPSCs, Arbas cashmere goats, RNA-sequencing

## Abstract

The Arbas cashmere goat is a unique biological resource that plays a vital role in livestock husbandry in China. LCDM is a medium with special small molecules (consisting of human LIF, CHIR99021, (S)-(+)-dimethindene maleate, and minocycline hydrochloride) for generation pluripotent stem cells (PSCs) with bidirectional developmental potential in mice, humans, pigs, and bovines. However, there is no report on whether LCDM can support for generation of PSCs with the same ability in Arbas cashmere goats. In this study, we applied LCDM to generate goat induced PSCs (giPSCs) from goat fetal fibroblasts (GFFs) by reprogramming. The derived giPSCs exhibited stem cell morphology, expressing pluripotent markers, and could differentiate into three germ layers. Moreover, the giPSCs differentiated into the trophectoderm lineage by spontaneous and directed differentiation in vitro. The giPSCs contributed to embryonic and extraembryonic tissue in preimplantation blastocysts and postimplantation chimeric embryos. RNA-sequencing analysis showed that the giPSCs were very close to goat embryos at the blastocyst stage and giPSCs have similar properties to typical extended PSCs (EPSCs). The establishment of giPSCs with LCDM provides a new way to generate PSCs from domestic animals and lays the foundation for basic and applied research in biology and agriculture.

## 1. Introduction

The Arbas cashmere goat is an excellent livestock breed in the Inner Mongolia Autonomous Region of China, which produces high-quality cashmere and meat and thus possesses significant economic value. In addition, goats can be used as an animal model for studying human disease and performing preclinical tests because of their strong similarity with the human genome [1,2].

Pluripotent stem cells (PSCs), which are isolated directly from blastocysts or induced from somatic cells, are characterized by the ability to self-renew and the potential to differentiate into all cell types in the organism [3,4,5]. PSCs hold great promise for basic biomedical research, the production of genetically modified animals, and clinical applications [6]. In animal husbandry, the PSCs of livestock such as goats can be used as good carriers for gene editing, effectively promoting excellent breed production [7,8]. Goat PSCs have been generated by optimizing the culture and somatic cell reprogramming strategies of human and mouse PSCs [1,9,10,11,12]. However, the proliferation capacity of goat PSCs is limited, and none of them produce germline chimeras. These goat PSCs are limited in their application to genetic epidemiology, disease models, and animal genetic breeding. Improving the quality of goat PSCs is essential to expanding their applications. Although PSCs have excellent developmental potential for all embryonic derivatives, their contribution to extraembryonic tissue is limited, especially to trophectoderm (TE) cells that develop into the placenta [13]. Recently, extended PSCs (EPSCs), a new type of PSC with embryonic and extraembryonic developmental potential, have been established [14,15,16]. EPSCs can be isolated from embryos or derived by somatic cell reprogramming [15,16]. EPSCs were first established using a chemical cocktail medium of recombinant human LIF, CHIR99021, (S)-(+)-dimethindene maleate, and minocycline hydrochloride (LCDM) [16]. Another culture system also has been used to derive mouse EPSCs from individual 8-cell blastomeres, which can develop into embryos and TE lineages in chimeras [14]. This system is also suitable for establishing human and porcine EPSCs [15]. In addition, a new type of chemically defined culture medium (the ABCL culture system) has been used to reprogram mouse epiblast stem cells (EpiSCs) into new embryonic stem cell lines (EpiSC-ASCs) with expanding potential [17]. In sum, the system of culturing EPSCs is widely used to derive EPSCs from various species, such as mice, humans, pigs, and bovines. Yet, whether goat EPSCs with great developmental potential can be derived using chemical cocktail culture remains unknown.

LCDM was used to generate EPSCs in mice [16], humans [16], and pigs [18]. Our previous report showed bovine stable induced PSCs (iPSCs) can be generated using LCDM, which possesses the characteristics of EPSCs and gives rise to both embryonic and extraembryonic tissue in vivo, which indicates that LCDM can be applied in high-quality PSC generation in domestic animals [19]. In this study, we obtained two giPSC lines by somatic reprogramming using LCDM. These giPSCs were stably maintained over a long term in culture and differentiated into three germ layers in vitro and in vivo. The giPSCs also had the potential to differentiate into the TE lineage. It is significant that giPSCs can contribute to embryonic and extraembryonic tissue in goat–mouse chimeras. The similarities and differences in molecular characteristics across goat iPSCs, bovine, human, and mouse EPSCs by LCDM were also investigated by RNA-sequencing (RNA-seq). We found that PSCs from different species had similar molecular features. Compared to goat preimplantation embryos, giPSCs showed similarities with goat blastocysts. Our study lays the foundation for mechanism study on goat iPSCs and promotes the use of goats in the fields of biology, agriculture, and medicine.

## 2. Results

### 2.1. LCDM Supports the Generation of giPSCs through Somatic Cell Reprogramming

**Figure 1:**
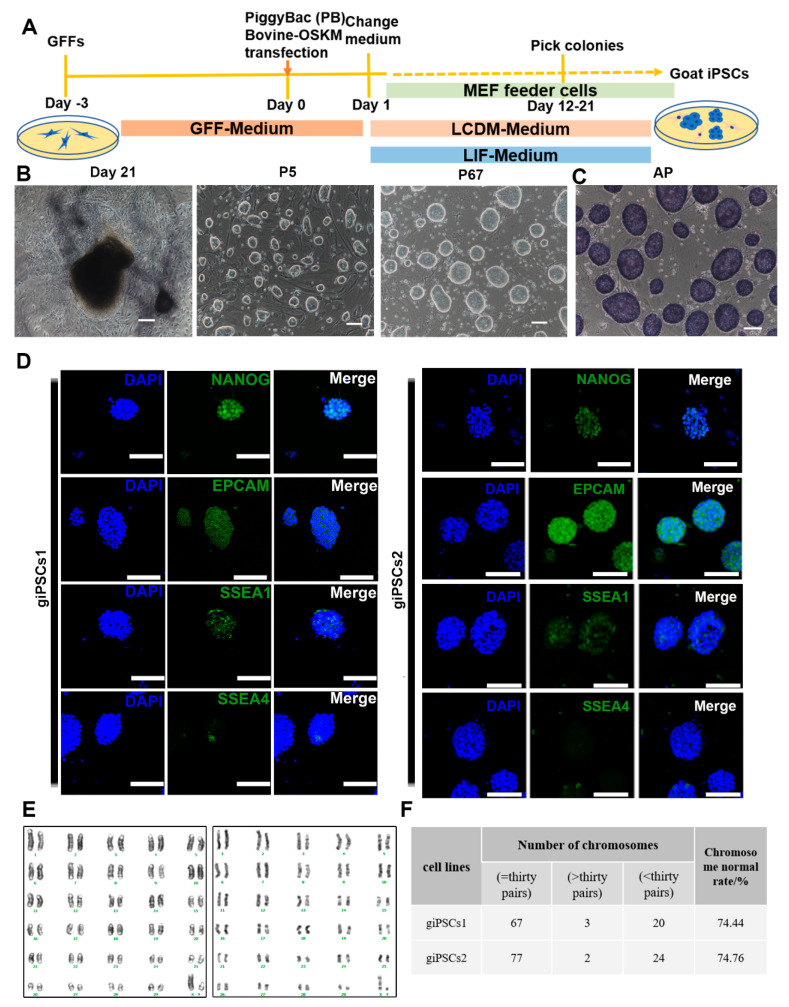
Derivation and characterization of giPSCs. (**A**) Schematic diagram of the generation of giPSCs from GFFs by reprogramming. (**B**) Representative morphologies at different stages in the reprogramming process. Scale bars, 100 µm. (**C**) AP staining of giPSCs (*n* = 3). Scale bars, 100 µm. (**D**) Immunostaining of pluripotency markers of giPSCs (*n* = 3). Nuclei were stained with DAPI. Scale bars, 100 µm. (**E**) Pictures from typical karyotype analysis of giPSCs (giPSCs1, passage 27; giPSCs2, passage 30). (**F**) Karyotype analysis and statistics for giPSCs.

To generate giPSCs, we used OSKM factors to reprogram goat fetal fibroblasts (GFFs) (Figure 1A). The transfected cells were cultured in LCDM or medium with LIF only (used as control). The cell morphology began to change on the fifth day after transfection. The colonies were obvious on days 12–21 and showed a dome-shaped morphology with clear borders (Figure S1A,B). These colonies were picked, digested by TrypLE into single cells, and then each cell was placed into a well in a 96-well plate. A total of 24 colonies were picked in LCDM medium, and 18 cell lines could be passaged. Two cell lines, called giPSCs1 and giPSCs2, were used for subsequent study. We picked 19 clones in the LIF culture system and found that only one clone could continue to grow (Figure S1C), but the passage ratio was about 1:1 to 1:2. The differentiation cells appeared at passage 5, and cells completely differentiated at passage 9. In order to detect the reprogramming effects of the two culture systems, we performed alkaline phosphatase (AP) staining and found that the clones generated in the LCDM were AP positive (Figure S1D). However, AP staining for the LIF culture system was not uniform (Figure S1E), and AP staining was negative after 30 min staining, and some positive cells only appeared after 7 h (Figure S1F). In conclusion, goat iPSCs cannot be generated with LIF only. The giPSCs generated in LCDM could stably proliferate, passage every 2–3 days, and keep for more than 60 generations (Figure 1B). To further explore the pluripotency of the giPSCs, we performed immunofluorescence staining of pluripotent markers. We found that the giPSCs were not only AP positive (Figure 1C), but also expressed pluripotent markers such as NANOG, EPCAM, and SSEA1, and barely expressed SSEA4 (Figure 1D). We also performed karyotype analysis on giPSCs (giPSCs1, passage 27; giPSCs2, passage 30) and detected normal G-banding (Figure 1E). Approximately 74% of cells displayed a normal diploid chromosome number (2n = 60; Figure 1F). To detect the copy number of exogenous transcription factors, we used a mixture of piggyBac vectors diluted at a ratio of 1, 10, 100, 1000, and 10000 with 50 ng GFFs genomic DNA to generate a standard curve (Figure S2A–D). The copy numbers of *OCT4*, *SOX2*, *KLF4,* and *cMYC* were detected, and they are 1.69 ± 0.23 and 3.04 ± 0.17, 0.48 ± 0.37 and 0.34 ± 0.06, 1.24 ± 0.14 and 0.60 ± 0.04, 4.38 ± 0.14 and 3.28 ± 0.67 in gPSCs1 and gPSCs2, respectively (Table S1). In summary, LCDM medium can be used to derive giPSCs and maintain their pluripotency.

**Figure 2:**
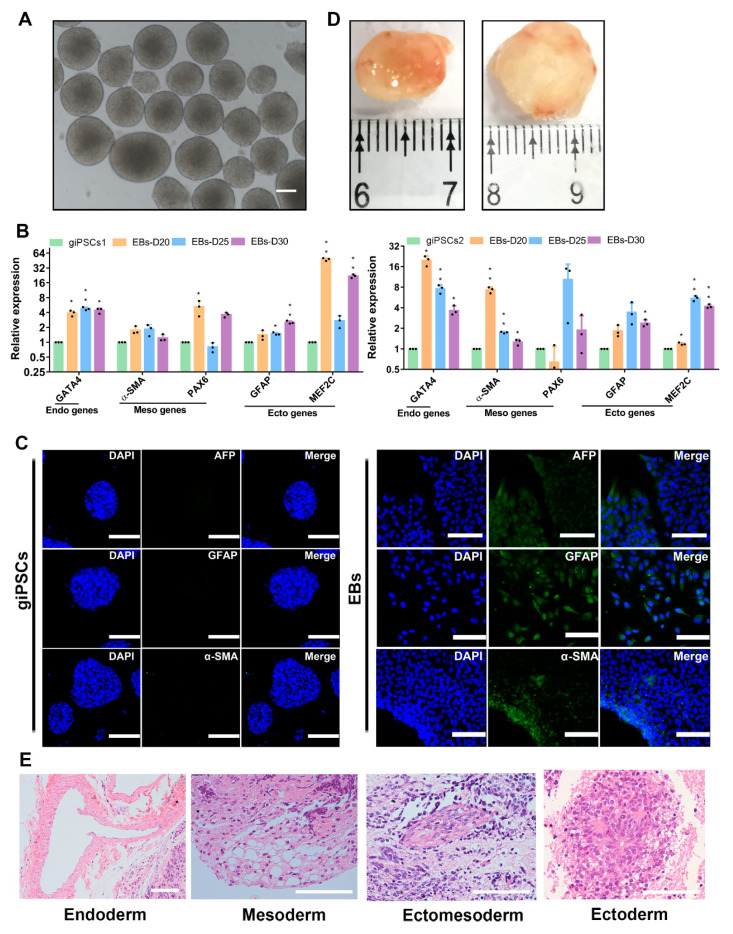
In vitro and in vivo differentiation of giPSCs. (**A**) EB morphologies (*n* = 3). Scale bars, 100 µm. (**B**) RT-PCR analysis of EBs. * *p* < 0.05; ** *p* < 0.01; *** *p* < 0.01. (**C**) Immunostaining of three germ layers (*n* = 3). Nuclei were stained with DAPI. Scale bars, 100 µm. (**D**) The teratoma differentiation of giPSCs in vivo. (**E**) H&E staining of teratomas. Scale bars, 100 µm.

To further analyze the differentiation potential of giPSCs, we performed embryoid body (EB) formation experiments in vitro and teratoma formation in vivo. We differentiated the giPSCs into EBs in the differentiation medium (Figure 2A). Real-time polymerase chain reaction (RT-PCR) analysis showed that the differentiated cells expressed endoderm gene *GATA4*, mesoderm gene *α-SMA* and *MEF2C*, and ectoderm genes *GFAP* and *PAX6* (Figure 2B). Immunofluorescence staining of glial fibrillary acidic protein (GFAP; ectoderm), actin smooth muscle (α-SMA; mesoderm), and a-fetoprotein (AFP; endoderm) indicated that the giPSCs could differentiate into three germ layers in vitro (Figure 2C). At the same time, we attempted to investigate whether giPSCs have the potential to produce PGCLCs in vitro, similar to mouse and human pluripotent stem cells. PGCLCs produced by giPSCs in embryonic bodies (EBs) (Figure S3A) were detected within 3–5 days, where early PGC genes such as *ITGB3*, *TFAP2C*, and *SOX17* were detected (Figure S3B). Through immunofluorescence staining, it was found that PGCLCs produced in embryonic bodies (EBs) express DAZL, DDX4, and PLZF proteins (Figure S3C). In addition, when giPSCs were injected into nude mice subcutaneously, they formed teratomas (Figure 2D). Hematoxylin and eosin (H&E) staining showed that teratomas have derivatives of three germ layers (Figure 2E). These results indicate that giPSCs have the ability to differentiate into three germ layers in vitro and in vivo.

### 2.2. giPSCs Have the Potential to Differentiate into the TE Lineages

**Figure 3:**
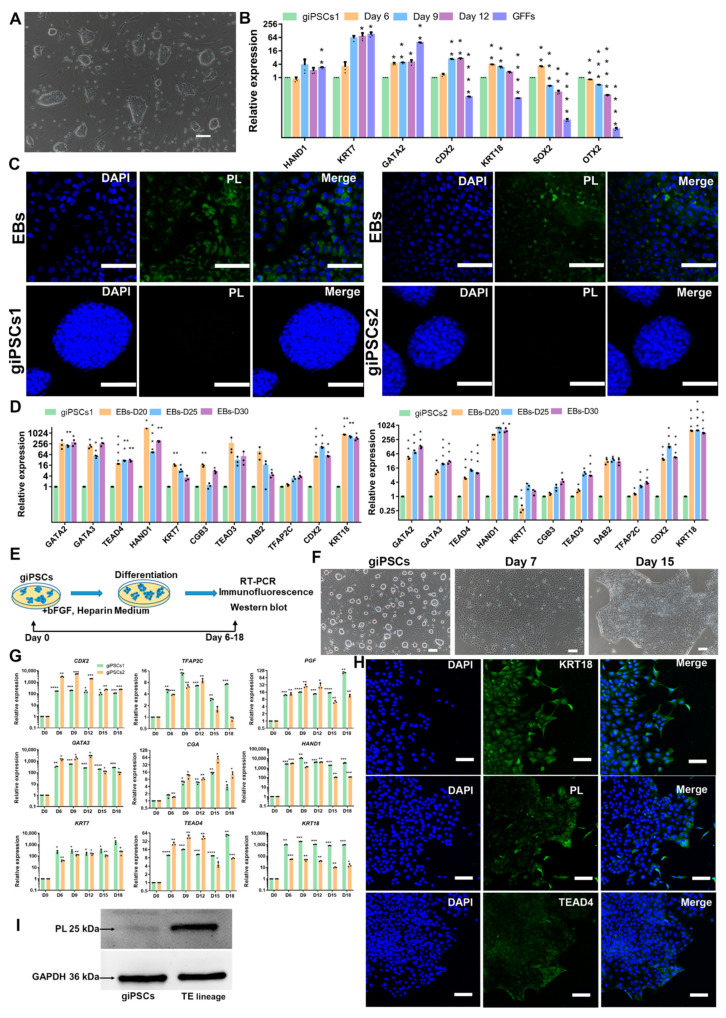
Differentiation of giPSCs into the TE lineage. (**A**) Representative image of giPSCs on day 6 after removal of the LCDM chemical cocktail. Scale bars, 100 µm. (**B**) Relative gene expression of differentiated giPSCs. * *p* < 0.05; ** *p* < 0.01; *** *p* < 0.001; **** *p* < 0.0001. (**C**) Immunostaining of EBs for PL (*n* = 3). Nuclei were stained with DAPI. Scale bars, 100 µm. (**D**) Relative expression of genes for the TE lineage in EBs. * *p* < 0.05; ** *p* < 0.01; *** *p* < 0.001; **** *p* < 0.0001. (**E**) Schematic diagram of the differentiation of giPSCs into the TE lineage in vitro. (**F**) Morphologies of the TE lineage from giPSCs. Scale bars, 100 µm. (**G**) Relative gene expression of TE marker genes. * *p* < 0.05; ** *p* < 0.01; *** *p* < 0.001; **** *p* < 0.0001. (**H**) Immunostaining of EBs (*n* = 3). Nuclei were stained with DAPI. Scale bars, 100 µm. (**I**) Western blotting analysis of PL protein.

TE is the first differentiated cell of mammalian embryogenesis and will develop into the placenta [20]. The potential to differentiate into TE lineage is an important characteristic of EPSCs, so we then investigated the TE differentiation ability of giPSCs by spontaneous differentiation and EB formation experiments. After the withdrawal of the chemical cocktail in the LCDM medium, the giPSCs began to differentiate (Figure 3A). Expression of pluripotency genes (including *SOX2*, *OTX2*) were downregulated and trophoblast markers such as *HAND1*, *KRT7*, *GATA2*, *CDX2*, and *KRT18* were upregulated (Figure 3B). At the same time, TE lineage can be detected in EBs. The trophoblast marker protein placental lactogen (PL) was expressed in EBs but not in giPSCs in the LCDM medium (Figure 3C). The expression of TE lineage genes such as *CDX2*, *TEAD4*, *KRT7*, *GATA3*, and *TFAP2C* were detected in EBs on day 20, day 25, and day 30 by RT-PCR (Figure 3D). Through FACS experiments, we found that the positive rate of TE-specific markers was about 20.6% (Figure S4A,B). These results indicate that giPSCs possess the differentiation ability into TE lineage.

To further assess the directed differentiation of giPSCs into the trophoblast lineage, we cultured giPSCs in trophoblast medium, which includes bFGF and heparin [21]. The morphology of the cells became flat on day 3 (Figure 3E,F). The expression of trophoblast genes such as *CDX2*, *TEAD4*, *KRT7*, *GATA3*, and *TFAP2C* were significantly upregulated, especially the trophoblast gene *CGA* (Figure 3G). The results of immunofluorescence staining showed that the trophoblast proteins KRT18, PL, and TEAD4 were expressed in the differentiated cells on day 12 (Figure 3H). Western blotting showed that the expression of PL protein was higher in the differentiated cells on day 12 than in giPSCs (Figure 3I). These results also indicate that giPSCs have the potential to differentiate into the TE lineage.

### 2.3. giPSCs Contribute to Embryonic and Extraembryonic Tissue in Chimeric Embryos

**Figure 4:**
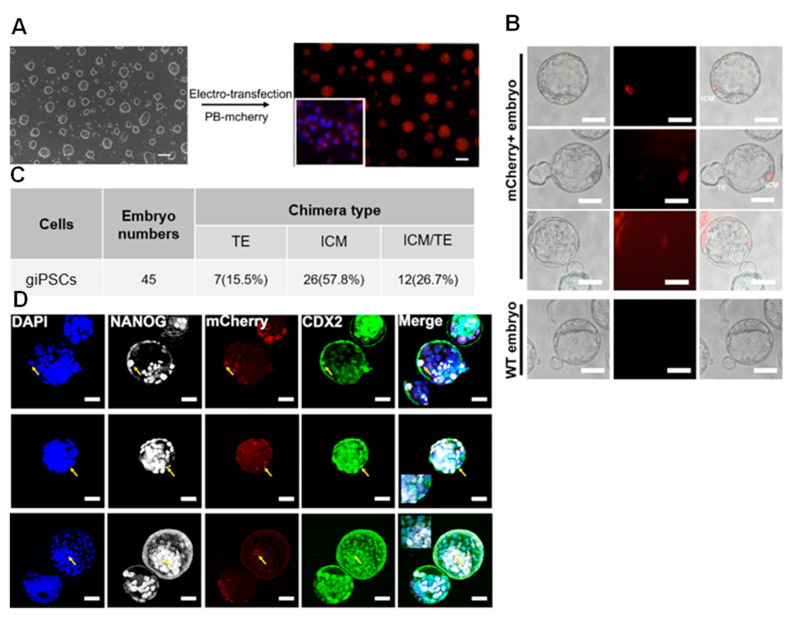
The giPSCs chimerism. (**A**) Bright field and mCherry of giPSCs. Scale bars, 100 µm. (**B**) Contribution of mCherry-labeled giPSCs to the TE and ICM in E3.5 chimeric embryos. Bar, 50 µm. (**C**) Summary of positive mCherry-labeled giPSCs. (**D**) Immunostaining of CDX2, NANOG, and mCherry in chimeric embryos. Bar, 50 µm.

To further explore the developmental potential of giPSCs, we microinjected giPSCs into mouse embryos. *PiggyBac* plasmids carrying the mCherry expression cassette were introduced into giPSCs to obtain the mCherry-labeled cells (Figure 4A). We injected 5–10 mCherry-labeled giPSCs into mouse embryos at the 4- to 8-cell stage and detected chimeric embryos at the blastocyst stage. The mCherry signals were detected both in the inner cell mass (ICM) and TE of chimeric embryos (Figure 4B,C). The results of immunofluorescence staining showed that mCherry colocalized with the ICM marker NANOG and the TE marker CDX2 in the chimeric embryos (Figure 4D), which indicates the contributions of giPSCs to embryonic and extraembryonic tissue in mouse blastocysts.

**Figure 5:**
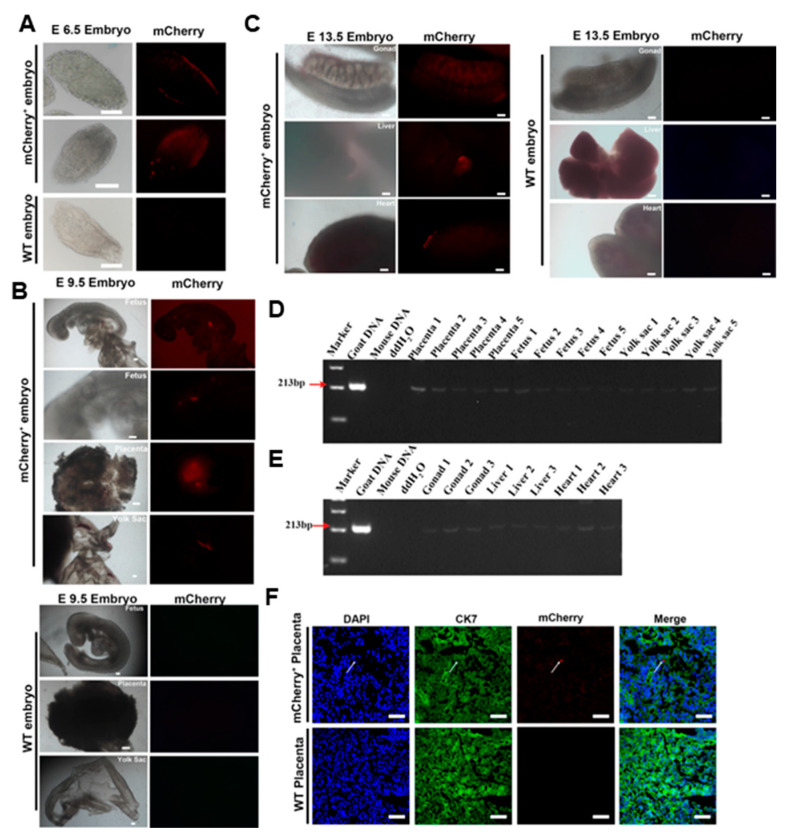
Contribution of giPSCs to embryonic and extraembryonic tissue in postimplantation goat–mouse chimeras. (**A**) Positive mCherry-labeled giPSCs were detected in E6.5 chimeric embryos. Bar, 100 µm. (**B**) giPSCs contributed to the fetus, yolk sac, and placenta in E9.5 chimeric embryos. Scale bars, 100 µm. (**C**) giPSCs contributed to gonad, liver, and heart tissue in E13.5 chimeric embryos (*n* = 3). Bar, 50 µm. (**D**) PCR analysis of the contribution of giPSCs in E9.5 embryos (*n* = 3). (**E**) PCR analysis of the contribution of giPSCs in E13.5 embryos (*n* = 3). (**F**) Immunofluorescence staining for CK7 and mCherry in the placenta (*n* = 3). Bar, 100 µm.

To explore the contributions of giPSCs to postimplantation chimeric embryos, we injected 5–10 mCherry-labeled giPSCs into mouse blastocysts, transferred the blastocysts into recipient mice, and detected the expression of mCherry in the chimeric embryos at E6.5, E9.5, and E13.5 (Table S2). Positive mCherry signals were detected in the E6.5 embryos (Figure 5A), and the chimeric contribution was about 25% (Table S2). At E9.5, mCherry signals were detected in embryonic and extraembryonic tissue, including the placenta and yolk sac of fetuses (Figure 5B). In addition, mCherry signals were also detected in the gonad, liver, and heart tissue of E13.5 chimeras (Figure 5C). Goat iPSCs can be detected in E13.5 chimeric gonadal tissue (Figure 5C) and have the potential to produce PGCLCs in vitro (Figure S3A–C), indicating that goat iPSCs have a certain contribution to the germ line. To further confirm the contribution of giPSCs, PCR was performed to detect specific sequences of goat mtDNA. Goat and mouse DNA were used as positive and negative controls, respectively. As expected, goat-specific mtDNA was detected in E9.5 chimeric fetuses and extraembryonic tissue (placenta and yolk sac; Figure 5D). Goat-specific mtDNA was also detected in the gonad, liver, and heart tissue of E13.5 chimeras (Figure 5E). Then, the chimeric placenta was immunostained with the placenta-specific marker (cytokeratin 7 [CK7]) and mCherry. The mCherry-positive placenta also exhibited CK7 positive, which indicates that giPSCs contribute to the placenta (Figure 5F). In these experiments, we found that the chimeric level of giPSCs in mice is very low. To evaluate whether the very low chimerism level is due to interspecific incompatibility, we tested cell fusion between goats and mice, and cultured mouse ESCs and giPSCs in LCDM. The results showed that the fusion rate of goat and mouse cells was very low at passage 4 (3.4%), and giPSCs were almost not detected at passage 9 (Figure S5A,B), which indicates the survival of giPSCs is difficult when cocultured with mouse cells. In sum, giPSCs contributed to embryonic and extraembryonic tissue in postimplantation goat–mouse chimeras, but the contribution is limited.

### 2.4. giPSCs Resemble Goat Blastocysts and Differ from GFFs and EPSCs of Other Species

**Figure 6:**
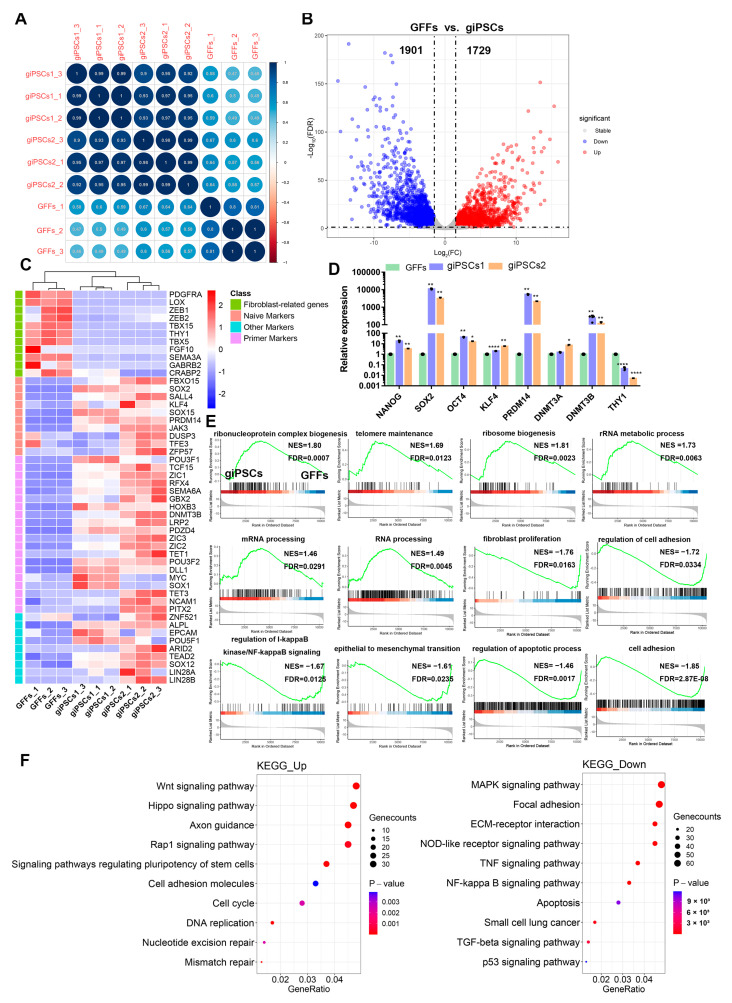
Transcriptomic features of giPSCs are different from those of GFFs. (**A**) Pearson correlation between giPSCs and GFFs. (**B**) The volcano plot between giPSCs and GFFs. (**C**) Heatmaps of giPSCs and GFFs. (**D**) RT-PCR analysis of pluripotent genes (*n* = 3). * *p* < 0.05; ** *p* < 0.01; **** *p* < 0.0001. (**E**) GSEA of giPSCs and GFFs. (**F**) KEGG pathways of DEGs between giPSCs and GFFs.

To further study the molecular characteristics of giPSCs, we collected giPSCs and GFFs for RNA-seq analysis. Bioreduced Pearson correlations showed a strong correlation at each stage (Figure 6A), which indicates that the RNA-seq data were highly repetitive. All differentially expressed genes (DEGs) were screened in giPSCs expression profiles. Compared to GFFs, 1729 and 1901 genes were upregulated and downregulated, respectively, in giPSCs (Figure 6B). Compared to GFFs, fibroblast-related genes (i.e., *LOX*, *ZEB1*, *THY1*, and *TBX5*) and pluripotent genes (i.e., *SOX2*, *JAK3*, *PRDM14*, and *SOX15*) were downregulated and upregulated, respectively, in giPSCs (Figure 6C). RT-PCR confirmed the RNA-seq results (Figure 6D). To determine the function of the DEGs, we performed Gene Set Enrichment Analysis (GSEA) and Kyoto Encyclopedia of Genes and Genomes (KEGG) enrichment analysis. Compared to GFFs, the upregulated genes in giPSCs were related to telomere maintenance, ribosome biogenesis, rRNA metabolic processes, mRNA processing, and ribonucleoprotein complex biogenesis in GSEA; the downregulated gene sets included fibroblast proliferation, regulation of I-kappaB kinase/NF-kappaB signaling, epithelial to mesenchymal transition, and regulation of apoptotic process and other signaling pathways (Figure 6E). KEGG pathway enrichment analyses showed that the upregulated signaling pathways in the giPSCs included Wnt signaling, the cell cycle, the pluripotency of stem cells, and DNA replication. The downregulated signaling pathways included the mitogen-activated protein kinase (MAPK), apoptosis, P53, and other signaling pathways (Figure 6F).

**Figure 7:**
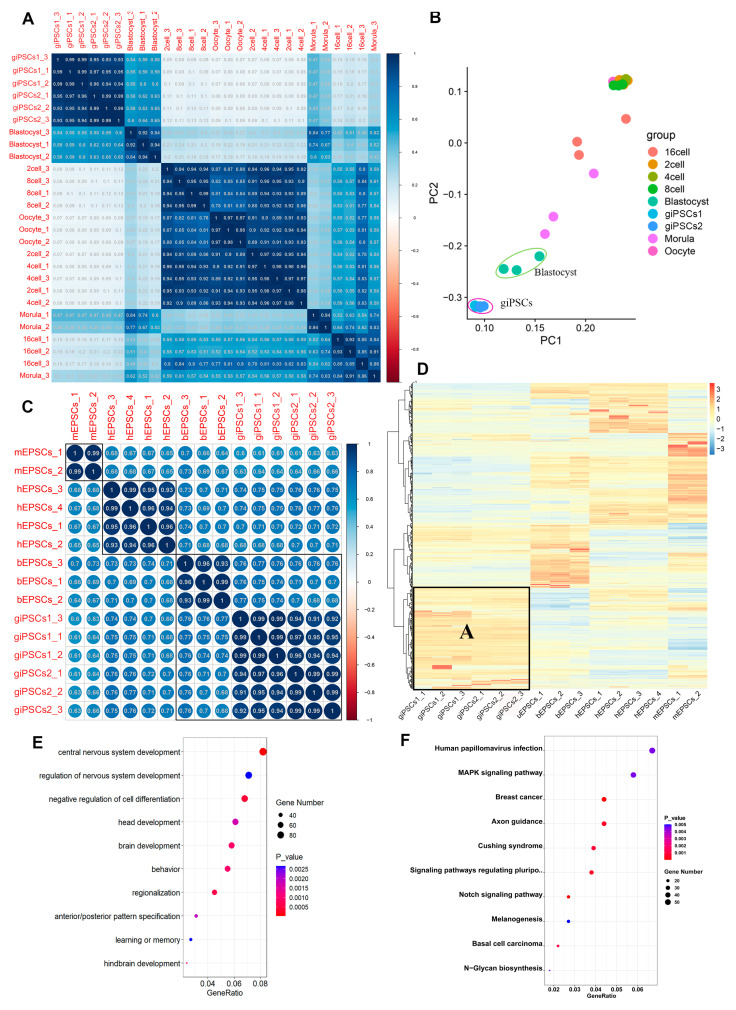
giPSCs resemble goat blastocysts and display unique characteristics. (**A**) Pearson correlation between giPSCs and preimplantation embryos. (**B**) PCA between giPSCs and preimplantation embryos. (**C**) Pearson correlations between mouse, human, and bovine EPSCs and giPSCs. (**D**) Heatmaps showing mouse, human, and bovine EPSCs and giPSCs. (**E**) GO analysis of DEGs between giPSCs and mouse, human, and bovine EPSCs. (**F**) KEGG pathway of DEGs between giPSCs and mouse, human, and bovine EPSCs.

We used Pearson correlation analysis and principal component analysis (PCA) to analyze the RNA-seq data of giPSCs and preimplantation goat embryos [22]. The giPSCs were more similar to goat blastocysts than embryos in the earlier stages (Figure 7A,B). We also analyzed the RNA-seq data of mouse EPSCs [16], human EPSCs [16], bovine EPSCs [19], and giPSCs generated in the LCDM culture system. The EPSCs of different species were strongly correlated, and the giPSCs and bovine EPSCs were closely related (Figure 7C). We next studied the DEGs of EPSCs of different species (Figure S6A and Figure 7D). Compared to mouse and bovine EPSCs, 2062 and 3019 genes were upregulated and downregulated, respectively, in giPSCs (Figure S6A). At the same time, the giPSCs exhibited unique gene expression profiles. Module A represented genes upregulated in giPSCs, which were unique to giPSCs and mainly participated in the regulation of nervous system development, brain development, and learning or memory (Figure 7D,E). KEGG enrichment signaling pathways were mainly focused on the MAPK signaling pathway, the Notch signaling pathway, and signaling pathways regulating stem cells (Figure 7F). Across the four species, EPSCs showed similar expression in placenta-related genes (including *SCD1*, *ITGA5*, and *TFAP2C*), genes encoding enzymes for DNA methylation (including *TET1*, *TET2*, *DNMT1*, *DNMAT3A*, and *DNMAT3B*), pluripotency genes (such as *POU5F1*, *SALL4*, *STAT3*, and *ZIC3*), and three germ layer markers (such as, *CDX2*, *PAX6*, *SOX17*, *ELF5*, and *GATA6*; Figure S6B–D). In short, the gene expression of giPSCs is close to goat blastocysts and exhibits unique molecular features compared with EPSCs from other species.

## 3. Discussion

EPSCs have been established in mice and humans [14,15,16], yet it is still challenging to establish comparable EPSCs in large livestock such as goats. Bovine EPSCs have been successfully established, which can proliferate stably for a long time and can differentiate into three germ layers in vitro. In chimeras, bovine EPSCs contribute to embryonic and extraembryonic tissue [19]. Furthermore, bovine EPSCs effectively achieve precise gene editing, and genetically modified bovine EPSCs can be used as donors for somatic cell nuclear transfer [23]. So far, goat EPSCs have not yet been established. In this study, we applied the LCDM culture system to establish giPSCs through reprogramming of GFFs. However, we found that all the differential markers expressed in EBs are comparable with giPSCs (<60 fold). In the EBs differentiation experiments of porcine EPSCs, the gene expression levels of each germ layer were all lower than 60 fold [15]. Similarly, we found that the expression levels of most germ layer genes were lower than 60 fold in bovine EB differentiation experiments [23]. Our findings are consistent with these findings, but giPSCs have the ability to differentiate into the three germ layers. The derived giPSCs maintained the characteristic of pluripotency and contributed to embryonic and extraembryonic tissues in preimplantation blastocysts and postimplantation chimeric embryos. RNA-sequencing analyses showed that the giPSCs were very close to goat blastocyst, and possessed similar properties to typical EPSCs. Furthermore, giPSCs were closer to bovine EPSCs but exhibited unique molecular features compared with EPSCs from other species. Although the established giPSCs exhibited pluripotency characteristics like mouse and human PSCs, the expression level of OCT4 and NANOG was relatively low in giPSCs. Similar results were also found in bovine EPSCs, in which the expression of NANOG was also about 100 fold higher in iPSCs than in fibroblasts [23]. Nanog, SoxB1, and Oct4 (Pouf1) activate transcription in mammalian preimplantation embryos and may play a role in mouse zygotic genome activation (ZGA) [24]. However, the timing of ZGA varies by species, with ZGA starting at the 1- to 2-cell stage in mouse embryos [25], at the 4- to 8-cell stage in humans and bovines [26,27], and at the 16-cell stage in goats [22]. Through embryonic transcriptome analysis, we found that the expression trends of Oct4 and Nanog in embryos varied by species (Figure S7A–C). Therefore, the pluripotency markers of PSCs of ruminants such as bovine and goats may be different from those of rodents and primates.

The extraembryonic differentiation ability is the main feature of EPSCs. Compared to traditional PSCs, EPSCs can differentiate into TE cells or TSCs [15,23]. Long-term overexpression of transcription factors (TFs) reprograms ESCs into trophoblast stem cells (TSCs) in vitro [20,28]. iPSCs, induced TSCs, and induced extraembryonic endoderm stem cells have been obtained by overexpressing TFs and then cultured in a suitable growth medium [29]. In this study, the giPSCs differentiated into the trophectoderm lineage by spontaneous and directed differentiation in vitro without overexpression exogenous factors. By evaluating the differentiation potential of giPSCs into the TE lineage, we found that the giPSCs expressed some TE marker genes, such as *KRT7* and *CDX2* in LCDM. When the culture system supplements bFGF, giPSCs could differentiate into the TE lineage without overexpression of any of the trophoblast marker genes.

Germ-line transmission is a widely accepted standard for evaluating the pluripotency of PSCs [30]. We injected giPSCs into mouse embryos to generate goat–mouse chimeras, and then analyzed the fate of the giPSCs at different developmental stages. These giPSCs contributed to both the ICM and TE in goat–mouse embryos. Note that after further development in vivo, giPSCs labeled with mCherry were observed in E9.5 and E13.5 goat–mouse chimeric placentas. However, the mCherry signal was not detected in the control group. In addition, giPSCs were found in the heart, liver, and gonad tissue in chimeric embryos at E13.5 but not in germ cells. It has been reported that low levels of chimerism have been observed between evolutionarily distant species, even in the early developmental stages [31,32,33]. There are several hypotheses about the difficulties of producing interspecific chimerism, including the death of injected PSCs, the failure of differentiation, and the huge evolutionary difference between donor PSCs and host animal species. At the same time, differences in amino acid sequences between ligands and receptors, early post-implantation development, cell adhesion, developmental rate, cell cycle, and pregnancy length also play key roles in limiting the formation of interspecies chimera [34]. It is reported that human naïve PSCs have almost no chimeric contribution in mice, pigs, and even rabbit and monkey embryos [34]. However, Deng and colleagues described produced human–mouse chimeras using human cells cultured in an EPSCs culture medium [16]. In this study, we found that giPSCs exhibit weak mCherry signaling and limited chimeric ability in goat–mouse fetuses, which may be due to the environment of the mouse womb cannot sustain the vitality of giPSCs.

Totipotent stem cells of mice, which resembled 2- and 4-cell embryos, were obtained by suppressing spliceosomal function [35,36]. According to the RNA-seq results, Liu laboratory EPSCs (L-EPSCs) showed similar characteristics to mouse E4.5 epiblast (EPI) cells or ESCs cultured in 2i/LIF, whereas Deng laboratory EPSCs (D-EPSCs) were similar to E5.5 EPI cells or EpiSCs [36]. EPSCs are similar to late multipotent EPI rather than embryos at the earlier developmental stage [35,36]. Which stage of embryos are giPSCs close to? We performed RNA-seq and compared giPSCs to preimplantation goat embryos. We found that the giPSCs were very close to the blastocysts, which was consistent with the results for mouse EPSCs. Although giPSCs and blastocysts are transcriptionally similar, further study is needed to uncover the exact developmental identity of these cells.

In summary, giPSCs with bidirectional developmental potential have been generated in LCDM. These giPSCs share some common gene expression profiles with EPSCs from other species and have specific transcriptional characteristics. The generation of giPSCs provides a useful cellular tool for better understanding initial cell fates and opens up new opportunities in medicine, biotechnology, and agriculture.

## 4. Materials and Methods

### 4.1. Animal Experiments

All animal procedures were performed in accordance with the guidelines of the Animal Protection and Utilization Committee and approved by the Inner Mongolia University Committee (approval code: IMU-MOUSE-2019-022, approval date: 26 August 2019) for animal experiments. CD1 (ICR) mice were purchased from Beijing Vital River Laboratory Animal Technology. Mice were housed under a 12 h light/dark cycle at 22 °C [37].

### 4.2. Generation of giPSCs through the Reprogramming of Somatic Cells

GFFs (goat fetal fibroblasts) from Arbas cashmere goats were a kind gift from Dongjun Liu of Inner Mongolia University. The GFFs medium is DMEM (11965-092, Gibco, New York, NY, USA) supplemented with 20% fetal bovine serum (50325, FBS; Bovoge, Melbourne, MEL, AUS) and 1% penicillin–streptomycin (15140122, Gibco, New York, NY, USA). The GFFs were cultured in the 6 cm dishes under 5% CO_2_ at 38.5 °C, and the medium was changed every day. When the cell densities reached approximately 90%, the GFFs were passaged [38].

PiggyBac plasmids, including CAG-bovine *OCT3/4*, CAG-bovine *SOX2*, CAG-bovine *KLF4*, and CAG-bovine *c-MYC*, were gifts from Xihe Li of Inner Mongolia University. The *PiggyBac* plasmid and PiggyBac transposase vector [39] were co-transfected into the GFFs by electroporation (approximately 10^6^ cells per electro-transfection). The GFFs were then plated at a density of 5000 cells per well in 12-well plates seeded with mitomycin C-treated mouse embryonic fibroblast cells and cultured in DMEM medium supplemented with 20% FBS under 5% CO_2_ at 38.5 °C. The medium was switched to the LCDM medium a day later.

The LCDM medium was prepared using a previously reported method [16]. The LCDM medium contained equal amounts of DMEM/F12 (11330-033, Gibco, New York, NY, USA) and Neurobasal (21103-049, Gibco, New York, NY, USA) supplemented with 0.5% N2 supplement (17502-048, Gibco, New York, NY, USA); 1% B27 supplement (17504-044, Gibco, New York, NY, USA); 1% L-glutamine (Sigma-Aldrich, St. Louis, MO, USA); 1% nonessential amino acids (M7145, Sigma-Aldrich, St. Louis, MO, USA); 0.1 mM β-mercaptoethanol (Sigma-Aldrich, St. Louis, MO, USA); 1% penicillin–streptomycin (15140122, Gibco, New York, NY, USA); 5% knockout serum replacement (10828028, Gibco, New York, NY, USA); 10 ng/mL recombinant human LIF (300-05, Peprotech, Cranbury, NJ, USA); 1 µM CHIR99021 (HY-10182, MCE, Monmouth Junction, NJ, USA); 2 µM (S)-(+)-dimethindene maleate (1425, R&D Systems, Minneapolis, MN, USA); and 2 µM minocycline hydrochloride (HY-17412, MCE, Monmouth Junction, NJ, USA). The LIF medium contained equal amounts of DMEM/F12 (Gibco, New York, NY, USA) and Neurobasal (Gibco, New York, NY, USA) supplemented with 0.5% N2 supplement (Gibco, New York, NY, USA); 1% B27 supplement (Gibco, New York, NY, USA); 1% L-glutamine (Sigma, St. Louis, MO, USA); 1% nonessential amino acids (Sigma-Aldrich, St. Louis, MO, USA); 0.1 mM β-mercaptoethanol (Sigma-Aldrich, St. Louis, MO, USA); 1% penicillin–streptomycin (Gibco, New York, NY, USA); 5% knockout serum replacement (Gibco, New York, NY, USA); 10 ng/mL recombinant human LIF (300-05, Peprotech, Cranbury, NJ, USA). Two cell lines, called gEPSC1 and gEPSC2, were used for sequent experiments. giPSCs were passaged every 3–4 days, and the medium was changed every day.

### 4.3. AP Staining

We performed AP staining using an Alkaline Phosphatase Staining Kit (C3206, Beyotime Biotechnology, Shanghai, China) according to the manufacturer’s instructions. Briefly, giPSCs were fixed with 4% paraformaldehyde (PFA) for 15 min at room temperature and stained with an AP staining kit at 37 °C for at least 15 min. Then, after rinsing twice with DPBS, the cells were photographed using an inverted light microscope (Nikon, Tokyo, Japan).

### 4.4. Karyotype Analysis

According to the standard G-banding chromosome analysis [37], giPSCs were treated with KaryoMAX Colcemid Solution (Gibco) at a final concentration of 2 mg/mL for 3 h. The cells were trypsinized, centrifuged at 1500 rpm, and resuspended in prewarmed hypotonic KCl solution (0.075 M) for 30 min at 37 °C. Then, 1 mL ice-cold fixative (3:1 mixture of methanol: glacial acetic acid) was added slowly and the cells were centrifuged at 1500 rpm for 5 min. The cells were washed twice and resuspended in 1 mL ice-cold fixative. The cells were then dropped from a height of about 1 m onto cold glass slides. The glass slides were dried overnight at room temperature and then dried in an oven at 70 °C for 1 h. The slides were treated with trypsin for 53 s at 37 °C and stained with Giemsa stain solution at room temperature for 30 min for the G-banding. The slides were then ready for microscopic observation. At least 50 metaphase cells were analyzed.

### 4.5. EBs Formation and In Vitro Differentiation

To prepare the EBs, we digested giPSCs into single cells and suspended them in a low-adhesion dish with IMDM (12440-053, Gibco) supplemented with 15% FBS (Bovogen) under 5% CO_2_ at 38.5 °C. After 4–7 days, the EBs were transferred onto a gelatin-coated cover slide to adherent plates. The medium was changed every 2–3 days. After 15–35 days, markers of three germ layers were analyzed by immunocytochemistry and RT-PCR.

### 4.6. Immunofluorescence Staining

For immunofluorescence staining, cells were fixed with 4% PFA for 30 min at room temperature, permeabilized with 1% Triton X-100 for 30 min, blocked with 5% BSA for 1 h, and incubated with primary antibodies overnight at 4 °C. After being washed with DPBS, the samples were incubated with secondary antibodies for 1 h at room temperature. Cells were stained with DAPI for 3–5 min at room temperature. Finally, visualization was achieved using a confocal laser scanning microscope (Nikon).

The primary antibodies were as follows: anti-NANOG (1:200; 500-P236, Peprotech); anti-SSEA1 (1:200; MAB4301, Santa Cruz Biotechnology, Dallas, TX, USA); anti-SSEA4 (1:200; MAB4304, Santa Cruz Biotechnology, Dallas, TX, USA); anti-EpCAM (1:200; ab71916, Abcam, Cambridge, UK); anti-CDX2 (1:200; #MU392A-UC, Biogenex, San Francisco, CA, USA); anti-AFP (1:200; MAB1368, R&D Systems, Minneapolis, MN, USA); anti-alpha smooth muscle actin (1:200; ab5694, Abcam, Cambridge, UK); anti-glial fibrillary acidic protein (1:200; Z0334, Dako, Carpinteria, CA, USA); anti-KRT18 (1:200; F4772, Sigma-Aldrich, St. Louis, MO, USA); anti-PL (1:200; ab15554, Abcam, Cambridge, UK); anti-TEAD4 (1:200; 12418-1-AP, Proteintech, Rosemont, IL, USA); and anti-human CK7 (1:200; M7018, Dako, Carpinteria, CA, USA).

The secondary antibodies were as follows: goat anti-mouse IgG and IgM antibody (1:500; AP130F, Millipore, Burlington, MA, USA) and Alexa488 goat anti-rabbit IgG (1:500; A-21206, Life Technologies, Carlsbad, CA, USA).

### 4.7. RT-PCR

Total DNA was extracted with a Dneasy Blood & Tissue Kit (Tiangen Biotech, Beijing, China). RNA extraction was performed with an Eastep™ Super Total RNA Extraction Kit (LS1040, Promega, Madison, WI, USA) following the manufacturer’s instructions. Complementary DNA was synthesized with a PrimeScript^RT^ Reagent Kit with gDNA Eraser (RR047A, Takara, Kusatsu, Japan). RT-PCR reactions were performed with a 7500 Real-Time PCR System (ABI Biosystems, Waltham, MA, USA) with GoTaq^®^ qPCR Master Mix (A6002, Promega, Wisconsin, WI, USA). Gene expression was calculated with the 2^−ΔΔCT^ method and normalized to the housekeeping gene *GAPDH*. Data are shown as means ± standard deviations. The sequences of the primers used are shown in Table S3.

### 4.8. Detecting Copy Number of Transgenes in giPSCs by Absolute Real-Time Quantitative PCR

The relative quantitative method was used to calculate the transgene copy number of giPSCs1 and giPSCs2 (ΔCt method), as described [40]. First of all, the absolute quantitative standard PCR [40] is established. The Quantitative PCR reaction uses 7500 Real-Time PCR System (ABI Biosystems) with GoTaq ^®^qPCR Master Mix (Promega). GADPH was amplified by quantitative PCR reaction as endogenous control. The ΔCt (ΔCt = Ct_transgene_ − Ct_GAPDH_) was plotted with the log2 of the transgene copy of the corresponding standard sample, and the quantitative standard curve was drawn. The copy number of giPSCs1 is calculated by this formula. Each polymerase chain reaction was repeated 3 times, and its value was means ± standard error.

### 4.9. Teratoma Formation

Approximately 1 × 10^7^ cells in 200 µL DPBS were injected subcutaneously into a 5-week-old male NOD-SCID mice. The mice were euthanized, and teratomas were obtained when they were 1 cm in diameter (3–5 months). Then, the teratomas were embedded in paraffin, and H&E staining was performed.

### 4.10. Differentiation of giPSCs into the TE Lineage

The cells of giPSCs were dissociated with TrypLE and plated in 6-well plates (1 × 10^5^ cells/well) in the TE cell medium. The TE cell medium contained 30% RPMI1640 (01-100-1ACS, BI) medium (including 20% FBS, 1 mM Na-pyruvate (Sigma), 1% Pen/Strep, 50 mM β-mercaptoethanol, 25 ng/mL Human FGF-basic (Peprotech), and 1 mg/mL heparin (Sigma) and 70% conditional medium of mitomycin C-treated mouse embryonic fibroblast feeder cells under 5% CO_2_ at 38.5 °C [21]. Markers of TE cells were analyzed by immunofluorescence, RT-PCR, and Western blotting.

### 4.11. Western Blotting

Cells were collected and lysed with lysis buffer (Thermo Fisher Scientific, Waltham, MA, USA) supplemented with phenylmethylsulfonyl fluoride (Beyotime) on ice for 30 min. The supernatant was collected after centrifugation at 13,200 rpm for 5 min. The BCA colorimetric method was used to measure the protein concentration. The samples were boiled for about 10 min. The proteins were then separated by SDS-PAGE with 10% Bis-Tris gels (Bio-Rad, Hercules, CA, USA) and transferred to PVDF membranes. Membranes were blocked in 5% skim milk in TBST for 1.5 h and incubated with primary antibodies overnight at 4 °C. And then incubated with secondary antibodies at room temperature for 1 h. Target protein bands were visualized by enhanced chemiluminescence (Thermo Fisher Scientific) and detected by an imaging analysis system (Bio-Rad). The antibodies were as follows: anti-PL (1:100; ab15554, Abcam, Cambridge, US), anti-GAPDH (1:2000; 10494-1-AP, Proteintech, NJ, USA), and anti-rabbit IgG (1:3000; 7074S, Cell Signaling Technology, Danvers, MA, USA).

### 4.12. Collection and In Vitro Culture of Mouse Embryos

Embryo collection and culture were conducted as described previously [19]. D1 female mice were superovulated by intraperitoneal injection of 5 international units (IU) PMSG. After 46–48 h, the mice were injected intraperitoneally with 5 IU HCG and caged with male mice. We obtained embryos at the 2-cell stage by flushing the oviduct with M2 at E1.5. These embryos were washed in M2, transferred into 15 µL KSOM drops covered with mineral oil, and maintained at 37 °C with 5% CO_2_ in an incubator.

### 4.13. Microinjection of giPSCs into 4- to 8-Cell Embryos and Early Blastocysts and Detection of the Developmental Fate of giPSCs

The giPSCs were injected into early embryos as described [19]. Briefly, 5–10 mCherry-labeled giPSCs were injected into 4- to 8-cell embryos and blastocysts. The injected 4- to 8-cell embryos were cultured for 36 h to detect the developmental fate of giPSCs in mouse late blastocysts. The injected blastocysts were cultured for 2–10 h and transferred into the uteri of pseudopregnant mice 2.5 days post coitum (dpc) to detect the developmental fate of giPSCs in mouse postimplantation embryos. A total of 18 chimeric embryos were transferred to a pseudopregnant mouse. At E6.5, E9.5, and E13.5 pregnant female mice were sacrificed. Fetuses and extraembryonic tissue were separated and chimeric contribution was detected by fluorescence stereoscopic microscopy, PCR, and immunostaining.

### 4.14. Flow Cytometry

The cells were digested into single cells with TrypLE, and fixed with 4% PFA at 4 °C for 20 min, then washed with PBS 3 times, and the suspension was filtered through a cell filter (40 μm, BD Falcon, State of New Jersey, NJ, USA). Analyze the samples on the Beckman CytoFlex LX machine. FlowJo software (v10.6.2, Ashland) was used for data analysis.

### 4.15. Cell Fusion

Mouse ESCs carrying a green fluorescent protein gene were a gift from Xia Wu of Inner Mongolia University. In the cell fusion experiment, we cocultured giPSCs carrying a red fluorescent protein gene with mouse ESCs. At first, mouse ESCs and giPSCs were digested to obtain single cells, respectively, then mixed at a ratio of 1:1 and centrifuged to discard the supernatant. The mixed cells were placed in 50% PEG 1500 solution for 1 min and stirred occasionally [41,42]. Then, the supernatant of the cells was centrifuged in the LCDM culture medium. The precipitate was re-suspended in the LCDM culture medium and plated in the plate containing the feeder layer, and the fresh culture medium was changed every day. After being cultured for several days, the double-positive cells were detected by flow cytometry.

### 4.16. RNA-seq and Analysis

The RNA-seq library was prepared as previously reported [43]. Briefly, RNA integrity and the total amount of RNA were accurately detected with an Agilent 5400 system (Agilent Technologies, Santa Clara, CA, USA). RNA-seq libraries were generated with an NEBNext^®^ Ultra RNA Library Prep Kit for Illumina^®^ (NEB, Ipswich, MA, USA) following the manufacturer’s instructions. After the generated libraries were qualified, they were pooled and sequenced on an Illumina Novaseq platform with the 150 bp paired-end mode (sequenced by Novogene). To ensure the quality and reliability of the data analysis, it was necessary to filter the original data. This mainly included removing reads with adapters, ploy-N, and low quality. The clean data were used for the subsequent analysis.

Clean reads were counted and generated with featureCounts v2.0.1. The clean reads were then mapped to the goat genome using Hisat2 v2.2.1 software tools [44]. The read counts of each gene were calculated, and the expression of each gene was standardized with TPM. DEGs were computed with the edgeR package in R [45]. An adjusted *p* < 0.05 and |Log2 (fold change)| ≥ 1.5 difference expression were considered significantly enriched by DEGs. Pearson correlation analyses, heatmaps, PCA, and hierarchical clustering were performed in R (v4.0.4).

The enrichment analyses of the DEGs in Gene Ontology (GO), KEGG, and GSEA were implemented using the ClusterProfiler R package [46], which corrects for gene length bias. A corrected *p* < 0.05 after calibration was considered significantly enriched by DEGs. To compare transcriptome profiles among species, we obtained mouse [16], human [16], and bovine [19] EPSCs from previous studies. All EPSCs cultured in LCDM medium were derived from similar systems. Goat preimplantation embryo data were derived from published articles [22]. Because batch effects in RNA-seq data have obvious differences among species and studies, batch correction is essential in cross-species comparison. We converted the FPKM in the original article to TPM for subsequent analysis. The corrected data were used to perform PCA and unsupervised clustering in R. To clarify the differences in EPSCs among different species, we analyzed the differences in TPM data among EPSCs from three species (b = 1000, k = 500) using ROTS [47] in R, then screened genes with *p* < 0.001, which we considered to be specifically expressed.

### 4.17. Statistical Analysis

All experiments were performed with three biological and technical replicates. Graphical presentation and statistical analysis of the data were performed with GraphPad Prism 6.0 (GraphPad Software, San Diego, CA, USA). Data were represented as means ± standard deviations, and statistical significance was calculated with Student’s two-tailed *t*-test: * *p* < 0.05; ** *p* < 0.01; *** *p* < 0.001; **** *p* < 0.0001.

## Data Availability

Data reported in this paper will be shared by the lead contact upon request. This paper does not report the original code. Any additional information required to reanalyze the data reported in this paper is available from the lead contact upon request.

## Supplementary Materials

The following supporting information can be downloaded at: https://www.mdpi.com/article/10.3390/ijms241914728/s1.

## Abbreviations

OSKM: Oct4-Sox2-Klf4-c-Myc; EPSC: extended pluripotent stem cell; PSC: pluripotent stem cell; giPSCs: goat induced pluripotent stem cells; LCDM: recombinant human LIF, CHIR99021, (S)-(+)-dimethindene maleate, and minocycline hydrochloride; iPSC: induced pluripotent stem cell; AP: alkaline phosphatase; EB: embryoid body; RT-PCR: real-time polymerase chain reaction; H&E: hematoxylin and eosin; DEG: differentially expressed gene; PCA: principal component analysis; GO: Gene Ontology; KEGG: Kyoto Encyclopedia of Genes and Genomes; GSEA: Gene Set Enrichment Analysis; PL: placental lactogen; MAPK: mitogen-activated protein kinase; TE: trophectoderm; ICM: inner cell mass; EPI: epiblast; EpiSC: epiblast stem cell; FBS: fetal bovine serum; GFF: goat fetal fibroblast; CK7: cytokeratin 7; TF: transcription factor; TSC: trophoblast stem cell; PFA: paraformaldehyde; IU: international unit.
