# Supplementary material for: Derivation of Arbas Cashmere Goat Induced Pluripotent Stem Cells in LCDM with Trophectoderm Lineage Differentiation and Interspecies Chimeric Abilities
Source: Int J Mol Sci. 2023 Sep 29;24(19):14728. doi: 10.3390/ijms241914728 (PMC10572416; doi:10.3390/ijms241914728)
Supplement: Supplementary file 1 [file ijms-24-14728-s001.zip › ijms-2609787-Supplementary materials.pdf]

Derivation of Arbas cashmere goat induced pluripotent stem cells in LCDM with trophectoderm lineage differentiation and interspecies chimeric abilities

Fang Liu[1], Jing Wang[1], Yongli Yue[1], Chen Li[1], Xuemin Zhang[1], Jinzhu Xiang[1], Hanning Wang[*1] and Xueling Li[*1]

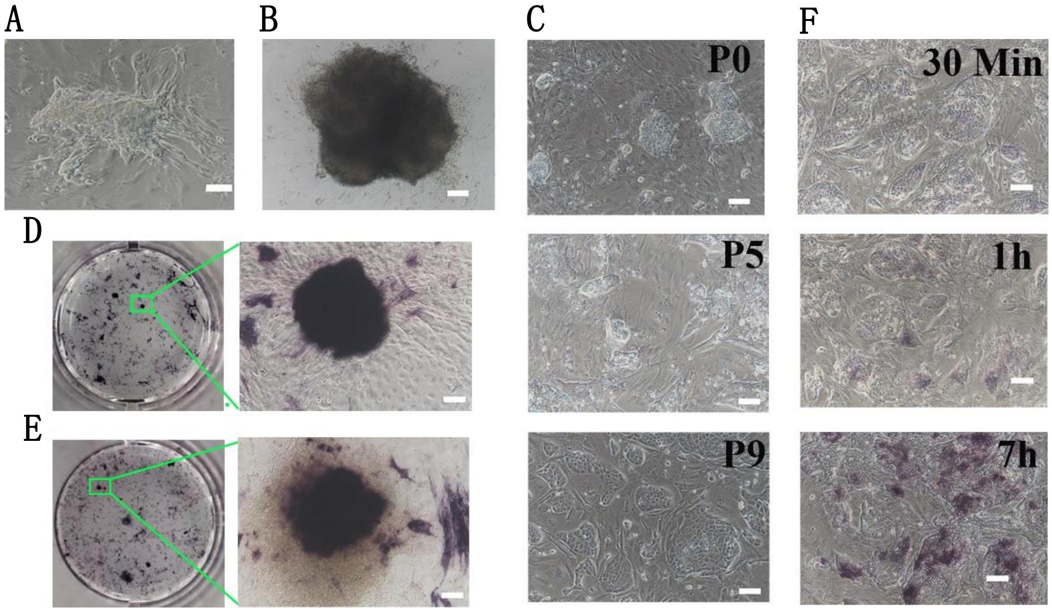

Figure S1. Use the LCDM and LIF culture medium to program the GFFs. (A) The reprogrammed GFFs on day 6. Scale bars, 100 μm. (B) The reprogrammed GFFs on day 21. Scale bars, 100 μm. (C) P0, P5, and P9 cells morphology of the LIF culture medium. Scale bars, 100 μm. (D) AP staining of LCDM culture medium (*n* = 3). Scale bars, 100 μm. (E) AP staining of the LIF culture medium (*n* = 3). Scale bars, 100 μm. (F) AP staining at different times for cells of the LIF culture medium.

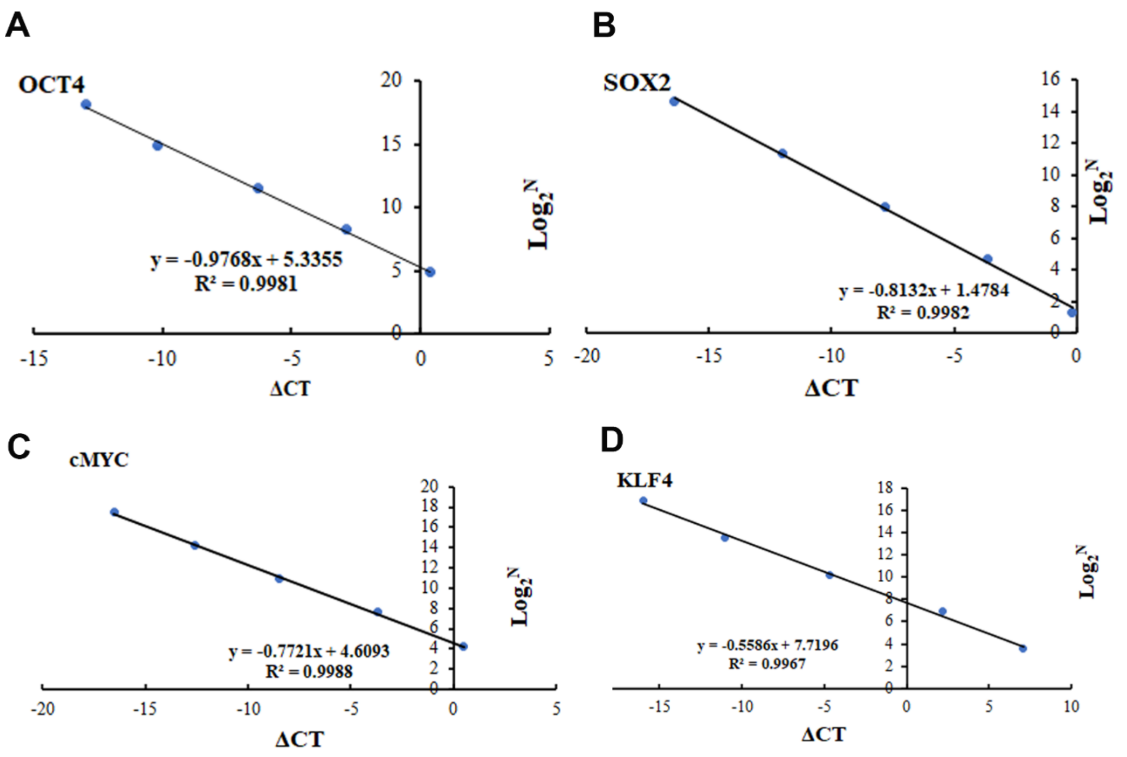

Figure S2. The copy number of the foreign genes in gEPSCs was analyzed by absolute quantitative PCR. (A) The absolute quantitative standard curves of transgenic SOX2. (B) The absolute quantitative standard curves of transgenic OCT4. (C) The absolute quantitative standard curves of transgenic KLF4. (D) The absolute quantitative standard curves of transgenic cMYC. The standard samples containing 1, 10, 100, 1000 and 10000 copies of the transgenes were prepared.

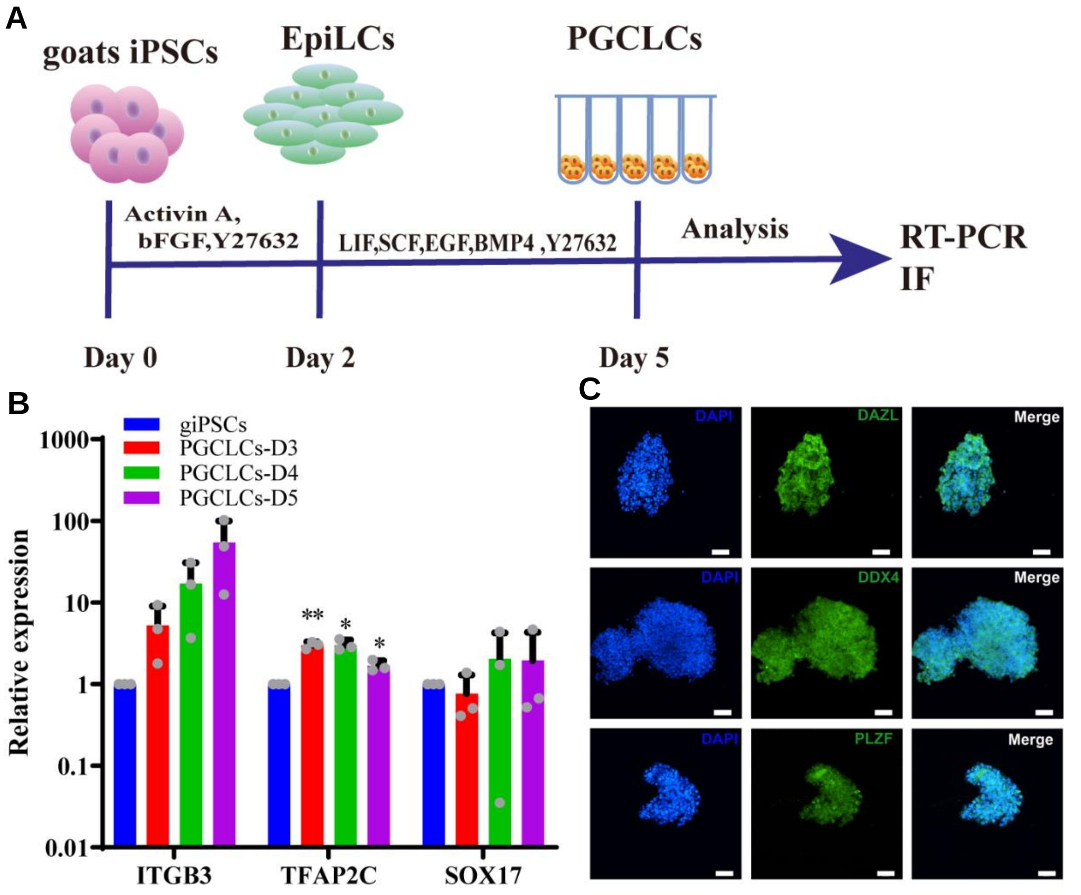

Figure S3. Contribution of giPSCs to embryonic and extraembryonic tissue in post-implantation goat-mouse chimeras. (A) Schematic illustration of giPSCs differentiation towards PGCLCs. (B) RT-PCR analysis of PGCLCs genes. *$p < 0.05$; **$p < 0.01$. (C) Immunostaining of markers of PGCLCs (*n* = 3). Nuclei were stained with DAPI. Scale bars, 100 μm.

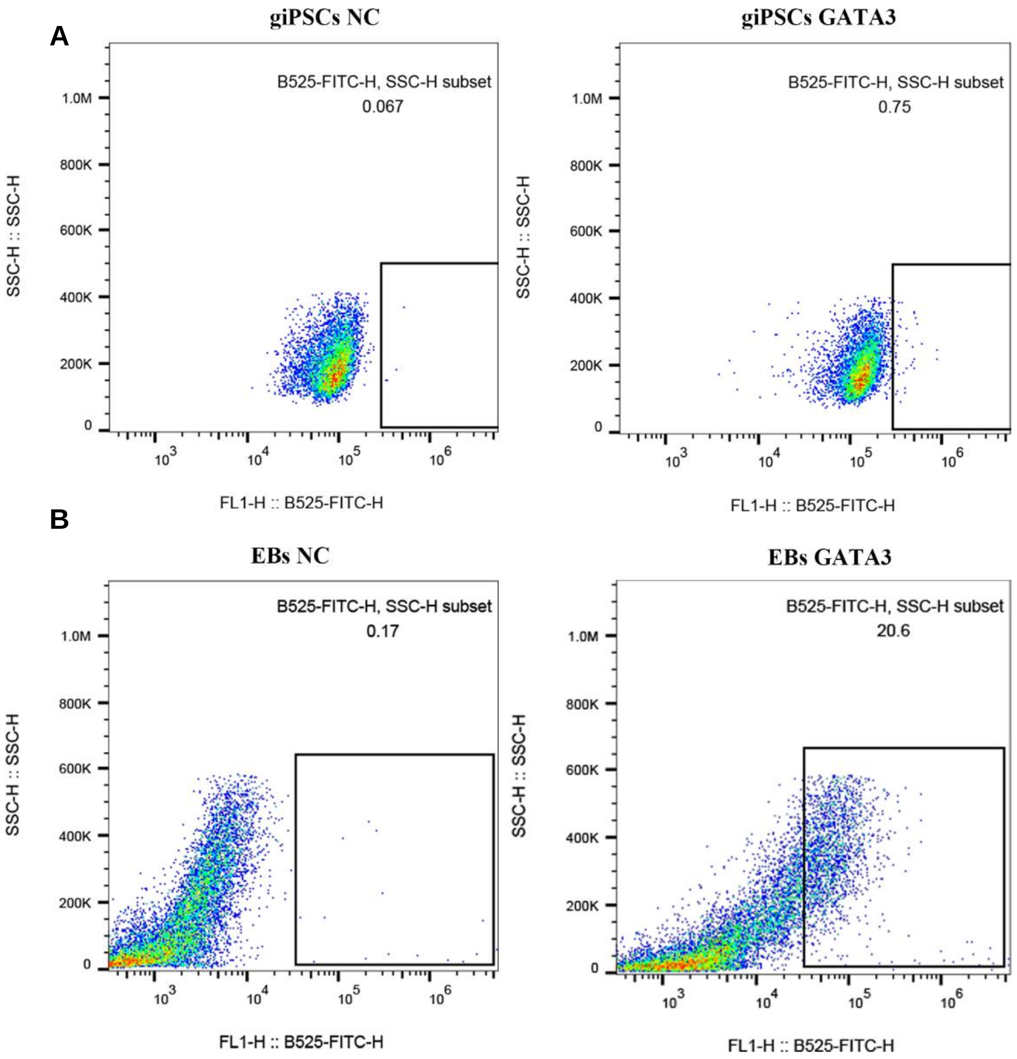

Figure S4. The flow cytometry analyses of TE markers. (A) The flow cytometry plot of intracellular GATA3 expression (right) and isotype control analysis (left) in giPSCs. (b) The flow cytometry plot of intracellular GATA3 expression (right) and isotype control analysis (left) in EBs.

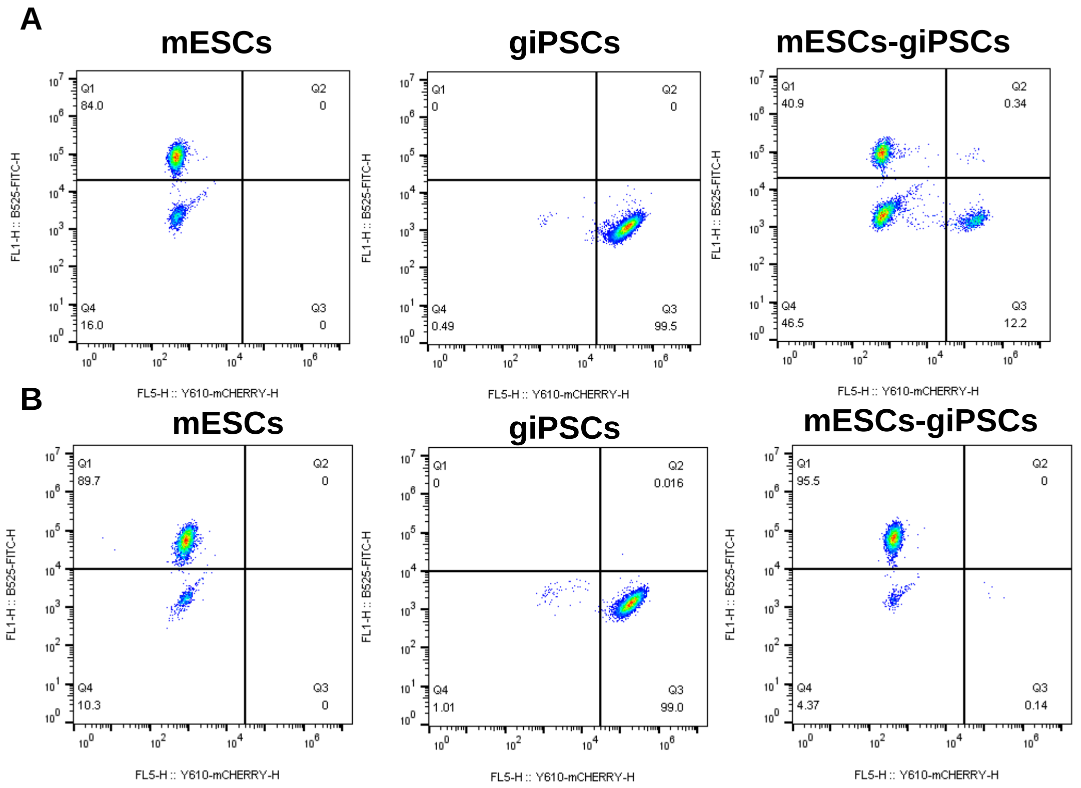

Figure S5. Cell fusion between mouse and goat cells. (A) FACS data with cell fusion between giPSCs and mESCs in P4. (B) FACS data with cell fusion between giPSCs and mESCs in P9.

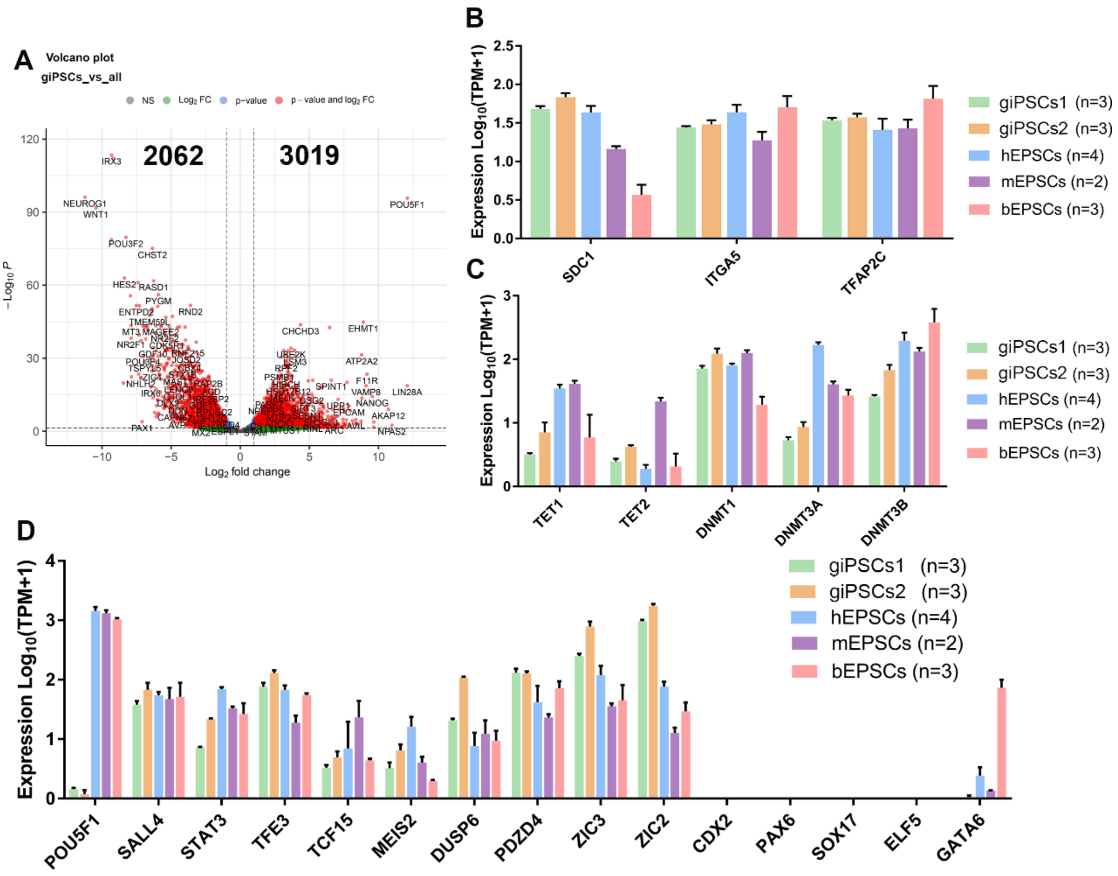

Figure S6. Global transcriptome profile of giPSCs. (A) The volcano plot between giPSCs, human EPSCs, mouse EPSCs, and bovine EPSCs. (B) Expression of lineage genes in human, mouse, and bovine EPSCs and giPSCs. (C) Expression of genes encoding enzymes for DNA methylation in human, mouse, and bovine EPSCs and giPSCs. (D) Expression of pluripotency genes in human, mouse, and bovine EPSCs and giPSCs. Data are means ± standard deviations.

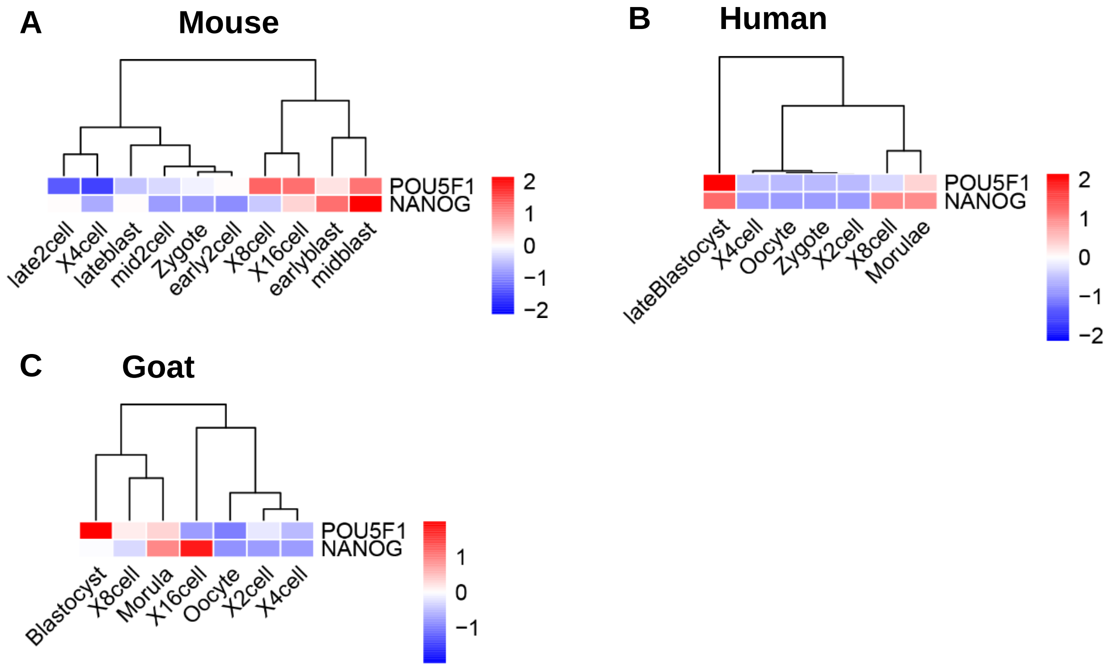

Figure S7. Heatmaps analysis of mouse, human, and goat embryos. (A) Heatmaps of mouse embryos. (B) Heatmaps of human embryos. (C) Heatmaps of goat embryos.

Table S1 The estimated copy numbers of transgenes by absolute real-time quantitative PCR.

Transgene	Cell Lines	Copy No. ±SD
OCT4	giPSCs1	1.69 ±0.23
	giPSCs2	3.04 ±0.17
SOX2	giPSCs1	0.48 ±0.37
	giPSCs2	0.34 ±0.06
KLF4	giPSCs1	1.24 ±0.14
	giPSCs2	0.60 ±0.04
cMYC	giPSCs1	4.38 ±0.14
	giPSCs2	3.28 ±0.67

Table S2 Summary of embryo injection

Chimera type	Cell numbers per embryo	Total no. of injected embryos	Total no. of embryos transplanted into recipient mice	Total no. of recipient mice	Total no. of pregnant mice	Total no. of fetuses	Total no. of mCherry positive embryos / Total no. of normal fetuses recovered at different stages		
							6.5 dpc embryos	9.5-10.5 dpc embryos	13.5 dpc embryos
Mouse blastocyst injection	5-10 giPSCs	224	189	9	7	55	4 / 16	6 / 21	7 / 18

Table S3 The primer sequences

Gene	NCBI accession number	Forward primer sequence (5′ to 3′)	Reverse primer sequence (5′ to 3′)
GAPDH	XM_005680968.3	CATCACTGCCACCCAGAAGAC	TCAGATCCACAACGGACACG
NANOG	NM_001314271.1	GATTCTTCCACAAGCCCT	TCATTGAGCACACACAGC
HAND1	XM_005683211.3	CGGCTGCAACTACACACTTC	CTTGGGTCCCACAGTCCTTC
α-SMA	XM_018065744.1	TGATGATTCTGATGTTTGGGATG	TGAAGCGATAGTAGCTGGGTAA
KRT18	XM_005680092.3	CCTGAGGGCTCAGATTTTTGC	CAGCCGGGTGACATTGGTAT
OTX2	XM_018054101.1	GGGCTGAGTCTGACCACTTC	GCGGCACTTAGCTCTTCGA
TEAD4	XM_018048627.1	GGCCACTACTCCTACCGAATCCA	TGCTCGCTCGCTGACACCTC
CGB3	XM_018062755.1	CCTGCCCTGTCTGTATCACTT	CCACGGGGAAAGAGACCATTG
TEAD3	XM_018038905.1	TGGGATCTGGAGAGGCTCAG	TTGGACGCTATTGTGCTGGT
DAB2	XM_018065603.1	TGTGACAGACAACCGAGCAT	CCATCTGGTCAACACCCAATTTC
PGF	XM_005686088.3	GACCAGCCCTTCTTTGTGGA	TGACCCTGGATCTCCTCCTTT
DNMT3B	XM_018057711.1	CGAGTATCAGGATGGGAAAGAGT	AGGTCGCCAGGTTAAAGTGC
THY1	XM_018059657.1	GAACCCTACCATTGGCATCG	GCACCCACTTCTTTGTATCACG
SOX2	NM_001285672.1	CGTGAACCAGCGGATGGACAGC	GGCCTGGAGTGGGAAGAAGAGGTA
OCT4	NM_001285569.1	AGAAGGGCAAACGATCAAGCAGT	TCAGGGAATGGGACCGAAGAGTA
KLF4	XM_018052560.1	TGCCAGAGGAGCCCAAACCAA	GCAAACTTCCACCCACAGCCATC
PRDM14	XM_018058124.1	GGTCAACACCAGCGAAGTCAA	GCTCACGGGAATGTCCAGAAA
DNMT3A	XM_018055548.1	CTGGCTCTTTGAGAATGTGGTGG	CCTCACTTTGCTGAACTTGGCTATT
GFAP	XM_018065254.1	CCTGCAGATCCGAGAAACCA	TAATGACCTCTCCATCCCGC
SMA	XM_018065744.1	TGATGATTCTGATGTTTGGGATG	TGAAGCGATAGTAGCTGGGTAA
GATA4	XM_018051833.1	CTTCTCAGGCTTAGGGAACATCA	GGGCACTGCTCCTTCTGGTAG
PAX6	XM_005690045.3	GTAGAACGCGGCTGTCAGAT	TGAGGCATCCTCTCTGGTCA

MEF2C	NM_001314204.1	CTGGGAGACCGTACCACCA	GGAGTGGAATTCGTTCCGGT
GATA2	XM_018046624.1	CCTCATCAAACCCAAGCGGA	GGTCAGAGGCCTGTTCACATTG
CDX2	XM_018056331.1	CTGTGCGAGTGGATGCGGAAGC	CGGATGGTGATGTAGCGACTGTAGTGA
GATA3	XM_018056970.1	CAAGGCGAGATCCAGCACAG	CAGGTAATGCCCGGTTCCAT
TFAP2C	XM_018057632.1	TTTCTCATTAAGGCGCCCCG	CCGCAATTGTAGATGCTGCC
CGA	XM_005684707.2	AGCATTTACCAAGGCCACAGT	TGGCAAGGAAAAGGAGGGTT
KRT7	XM_005680100.3	CTTCATCGACAAGGTGCGGT	AGCTCCACCTCTAGACGACC
B-OCT4	NM 174580.2	GGTTCTCTTTGGAAAGGTGTTC	ACACTCGGACCACGTCTTTC
B-SOX2	NM 001105463.2	CATCCACAGCAAATGACAGC	TTTCTGCAAAGCTCCTACCG
B-KLF4	NM 001105385.1	TCCCACCGCTCCATTAC	ATGAGAACTCTTCGTGTAGG
B-cMYC	NM 001046074.2	CCCATCAGCACAATTACGCA	TGTCCGCCTCTTGTCATTCT
